# COVID-19 and Heat Illness in Tokyo, Japan: Implications for the Summer Olympic and Paralympic Games in 2021

**DOI:** 10.3390/ijerph18073620

**Published:** 2021-03-31

**Authors:** Kazuki Shimizu, Stuart Gilmour, Hiromi Mase, Phuong Mai Le, Ayaka Teshima, Haruka Sakamoto, Shuhei Nomura

**Affiliations:** 1 Faculty of Public Health and Policy, London School of Hygiene and Tropical Medicine, London WC1E 7HT, UK; 2 Department of Health Policy, London School of Economics and Political Science, London WC2A 2AE, UK; 3 Graduate School of Public Health, St. Luke’s International University, Tokyo 104-0045, Japan; sgilmour@slcn.ac.jp (S.G.); lemaiphuong166@gmail.com (P.M.L.); 4 Department of Epidemiology and Public Health, Institute of Epidemiology and Health Care, University College London, London EC1E 7HB, UK; hiromi.mase.19@ucl.ac.uk; 5 Faculty of Medicine, School of Public Health, Imperial College London, London W2 1PG, UK; ayaka.teshima20@imperial.ac.uk; 6 Department of Health Policy and Management, School of Medicine, Keio University, Tokyo 160-8582, Japan; sakamotoh@keio.jp (H.S.); s-nomura@keio.jp (S.N.); 7 Department of Global Health Policy, Graduate School of Medicine, The University of Tokyo, Tokyo 113-0033, Japan

**Keywords:** COVID-19, pandemic, heat illness, mass gathering, health systems, health security, health services, risk assessment

## Abstract

The 2020 summer Olympic and Paralympic Games in Tokyo were postponed to July–September 2021 due to the coronavirus disease 2019 (COVID-19) pandemic. While COVID-19 has emerged as a monumental health threat for mass gathering events, heat illness must be acknowledged as a potentially large health threat for maintaining health services. We examined the number of COVID-19 admissions and the Tokyo rule for emergency medical care, in Tokyo, from March to September 2020, and investigated the weekly number of emergency transportations due to heat illness and weekly averages of the daily maximum Wet Bulb Globe Temperature (WBGT) in Tokyo in the summer (2016–2020). The peak of emergency transportations due to heat illness overlapped the resurgence of COVID-19 in 2020, and an increase of heat illness patients and WBGT has been observed. Respect for robust science is critical for the decision-making process of mass gathering events during the pandemic, and science-based countermeasures and implementations for COVID-19 will be warranted. Without urgent reconsiderations and sufficient countermeasures, the double burden of COVID-19 and heat-related illnesses in Tokyo will overwhelm the healthcare provision system, and maintaining essential health services will be challenging during the 2021 summer Olympic and Paralympic Games.

## 1. Introduction

The ongoing coronavirus disease 2019 (COVID-19) pandemic significantly affected the livelihood of citizens at an unprecedented scale, and second and third waves of the virus have shocked many countries. Although Japan has so far mitigated the worst epidemiological impact of COVID-19 relative to other high-income countries, weak coordination among health systems, an increase in nosocomial COVID-19 infections, insufficient financial assistance for healthcare institutions, and social discrimination against healthcare workers affected the maintenance of overall health services during the first wave of COVID-19 [1,2,3,4,5]. A resurgence of COVID-19 in Japan from July 2020 overlapped with the increasing demand for healthcare in the summer, especially due to heat illness. Insufficient preparedness for maintaining an effective reproduction number below 1 under the suppression strategy brought the second wave of COVID-19 [2], which consequently overstretched healthcare provision systems in the country at that time.

After originally being postponed from 2020 [6], the summer Olympic and Paralympic Games in Tokyo (Tokyo 2020) are scheduled in July–September 2021, but whether the games will be safely held under the ongoing COVID-19 pandemic, and how they should be organized, are becoming a critical agenda. While global attention has been paid to managing COVID-19, it should be noted that multiple health threats were recognized in the preparatory phase of Tokyo 2020 in the pre-COVID-19 era, and the weakness of health risk assessment for environmental conditions became evident [6]. As Tokyo 2020 was scheduled during the hottest and most humid period of the year in Japan, when the number of heat illness patients usually peaks [7,8,9], there was concern about securing sufficient healthcare resources for appropriately responding to healthcare demands [6]. Now, the COVID-19 pandemic has emerged as the largest public health threat associated with international mass gathering events (MGEs), Japan needs to address these multiple health threats simultaneously; however, as of today, scant research has addressed the cumulative burden caused by COVID-19 and other health threats, and filling this gap is critical.

This report describes how emergency medical services were affected by the COVID-19 pandemic, examines the previous trend in heat-related illness and meteorological data during the period of the summer Olympic and Paralympic Games in Tokyo and explores challenges and implications for this and other mass gathering events (MGEs) in the COVID-19 era.

## 2. Materials and Methods

We first compared the temporal trends in the number of hospital admissions for COVID-19 in Tokyo in March–September 2020, with the number of emergency hospital transportations rejected by more than 5 hospitals or requiring more than 20 min before finding an appropriate medical facility (the so-called “Tokyo Rule”). The Tokyo Rule was introduced in 2009 as a surrogate indicator of pressure on emergency medical services in Tokyo [10]. Both data were obtained from the open-access dashboard tracking the COVID-19 pandemic in real-time in Tokyo (called “Updates on COVID-19 in Tokyo”), which is administered by the Tokyo Metropolitan Government [11].

Second, to analyze the state of emergency transportations due to heat illness in Tokyo during the period when the next summer Olympic and Paralympic Games are to be held, the number of emergency transportations due to heat illness in the period was aggregated by age and severity over the past five years. This is also open access data available at the website of the Fire and Disaster Management Agency (FDMA), Ministry of Internal Affairs and Communications, Japan [12]. Because the games are scheduled to be held from 23 July to 5 September 2021, corresponding to weeks 29 to 36 of the epidemiological calendar as defined by the National Institute of Infectious Diseases [13], data from the same period in each of the past five years were considered. According to the FDMA, patients who only required outpatient care were defined as mild cases, with moderate cases requiring hospitalization for less than 3 weeks. Severe/fatal cases included patients who required hospitalization for 3 weeks or more and/or were confirmed dead. The trend in weekly averages of the daily maximum Wet Bulb Globe Temperature (WBGT) in Tokyo, a heat stress index commonly utilized in daily life, workplaces and for considering the re-scheduling or cancelling sporting events [14], was presented in line with epidemiological weeks mentioned above. WBGT is widely used as an indicator of risk of heat-related illness in Japan, and it is advised that all exercise must be prohibited when the WBGT is above 31 degrees Celsius (°C), and a WBGT of 28–31 °C and 25–28 °C are classified as “severe warning” and “warning”, respectively [15]. The Ministry of the Environment calculates WBGT as follows:

$$WBGT\;\left({}^{\circ}C\right)=0.7\times \mathrm{Tw}+0.2\times \mathrm{Tg}+0.1\times \mathrm{Ta}$$

where Tw, Tg, and Ta represent Natural Wet Bulb temperature, Globe Temperature and Natural Dry Bulb, respectively. Tw was calculated from the temperature, humidity, and atmospheric pressure data measured by the Japan Meteorological Agency; while Tg and Ta were directly collected by the Japan Meteorological Agency [16]. We calculated weekly averages of daily maximum WBGT from the hourly WBGT data published by the Heat Illness Prevention Information, Ministry of the Environment, Japan [17].

Finally, we explored minimum conditions and several risks for holding MGEs during COVID-19 by referring to a publicly available risk assessment tool [18], and summarized several challenges in simultaneously managing heat illness, COVID-19, and other essential health services during the next summer Olympic and Paralympic Games in Tokyo. 

## 3. Results

**Figure 1:**
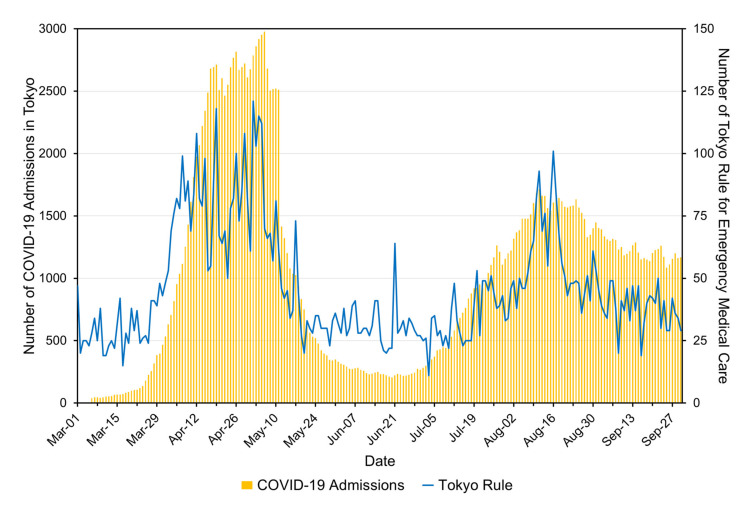
Number of coronavirus disease 2019 (COVID-19) admissions and Tokyo rule for emergency medical care in March–September 2020. Yellow bars illustrate the number of COVID-19 admissions, and the blue line presents the trend in the Tokyo rule for emergency medical care. Please note that the number of COVID-19 admissions before 11 May included patients isolated at designated accommodations or home, and the number of hospitalized COVID-19 patients before 5 March was not published on the dashboard [11].

Figure 1 shows the number of COVID-19 admissions (left axis) and the number affected by the Tokyo Rule for emergency medical care (right axis) in Tokyo in March–September 2020. In the early phase of the COVID-19 pandemic, the number of COVID-19 admissions dramatically increased from less than 100 before mid-March to a maximum of 2974 in early May. Before the surge in COVID-19 admissions, the number of Tokyo Rule transportations ranged between 20 and 40 per day, and this suddenly started to increase in early April. On 12 April, the daily number of Tokyo Rule transportations was recorded above 100 for the first time, and reached this peak in early May. In line with the decreasing trend in COVID-19 admissions, the daily number of Tokyo Rule transportations also started to decrease, and finally reached less than 20 in mid-June. When Japan faced the resurgence of COVID-19 from July 2020, the number of COVID-19 admissions again rose to above 1600 in August. In the period, the number of Tokyo Rule transportations gradually increased in early to mid-August with sudden rapid increases to 93 on 11 August (Week 33) and 101 on 16 August (Week 34), which corresponded to the period when admissions, due to heat illness, increased.

The number of emergency transfers due to heat illness, aggregated by year and age group, is summarized in Table 1. The cumulative number of emergency transportations during the study period increased from 1789 in 2016 to 6269 in 2018, followed by a decrease to 4862 in 2020. The proportion of heat illness transfers among those aged 65 and above has been on an increasing trend, and those aged 65 and over represent more than half of all transportations in 2019 and 2020. The proportion of patients who required admission, namely the cumulative proportion of moderate, severe and death cases among all transfers, changed from 38.1% in 2016 to 42.0% in 2019 and 42.7% in 2020, and severe/fatal cases occupied around 5% of all transfers after 2019.

**Figure 2:**
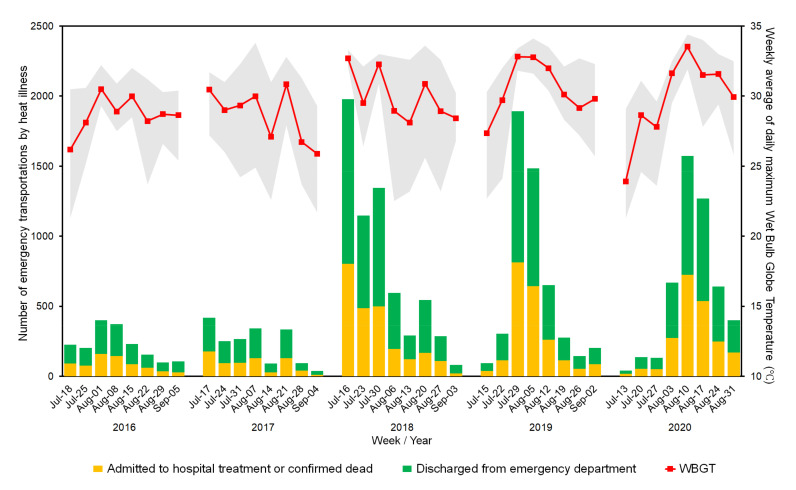
Number of emergency transportations due to heat illness by disposition and weekly averages of daily maximum Wet Bulb Globe Temperature (WBGT) in Tokyo in 2016–2020. Yellow indicates the number of heat illness patients requiring admission, or confirmed dead, while green shows mild cases of patients who did not need admissions. The red line shows the trend in the weekly averages of daily maximum WBGT. Gray-shaded areas represent the variation of daily maximum WBGT in each week.

Figure 2 presents emergency transportations due to heat illness (left axis) and weekly averages of daily maximum WBGT (right axis) in Tokyo in 2016–2020, by weeks and disposition. While the weekly number of heat illness emergency transportations was limited to less than 500 in 2016–2017, it rose to over 1500 in 16–22 July 2018 (week 29), 29 July–4 August 2019 (week 31), and 10–16 August 2020 (week 33). Emergency transportations were overcrowded for approximately 3–4 weeks in the recent 3 years: namely, 16 July–12 August 2018 (weeks 29–32); 29 July–18 August 2019 (weeks 31–33); and 3–30 August 2020 (weeks 32–35). Regarding ambient conditions, the weekly average of daily maximum WBGT first rose to above 31 °C in 16–22 July (week 29) and 30 July–5 August (week 31) in 2018. Afterwards, it increased to above 31 °C in 29 July–18 August 2019 (weeks 31–33) and 3–30 August 2020 (weeks 32–35), and reached a maximum of 33.5 °C in 10–16 August 2020.

## 4. Discussion

### 4.1. Emerging Health Threats in Tokyo: Heat Illness

While multiple health threats, especially infectious diseases, have been discussed in the preparatory phase of Tokyo 2020 [6,19,20,21,22], addressing the increasing number of patients suffering from heat illness in the summer season has been a critical agenda [7,8,9], because Tokyo 2020 was scheduled during the hottest and most humid period of the year in Japan, and the maximum air temperatures regularly exceeded 35 °C [23]. Although the government has started to introduce a new heatstroke alert system to raise citizens’ awareness in the summer 2020 [24], the extent to which heat illness would impact healthcare provision during MGEs, including the Olympic and Paralympic Games, was not sufficiently discussed.

The weekly data show that emergency transportations were centralized in around 3–4 weeks in the summer season, especially in 2018–2020, which coincided with the original Tokyo 2020 period. More than a half of all transportations were of people aged 65 and above, who have been recognized as high-risk group for hospitalization [25], suggesting an increasing impact of heat illness on hospital bed capacity, especially acute care beds. Although it is challenging to estimate to what extent the increase in COVID-19 cases and heat illness transportations contributed to the increase in Tokyo Rule transportations, this report presents clear evidence that emergency medical services faced difficulties in timely delivery of patients to appropriate healthcare institutions around early to mid-August 2020. Considering that excess/exiguous heat illness transportations were not observed in Tokyo in 2020 [26], it can be assumed that similarities of some initial clinical symptoms between COVID-19 and heat illness (i.e., fever, fatigue) [27] might have worked as challenges for ambulances to deliver patients to appropriate healthcare institutions in a timely fashion, thus increasing the number of Tokyo rule transportations; but reasons behind this must be closely investigated for ensuring patients’ fast access to emergency rooms and early intervention. Further, the weekly averages of daily maximum WBGT were first recorded above 31 °C in 2018, but remained above 31 °C for over 4 weeks in 2020 with a maximum of 33.5 °C in 10–16 August 2020 (week 33). It should be noted that the Ministry of Environment, Japan, declared that all exercise must be prohibited at a WBGT of 31 ℃ or above [15]. The trend in WBGT in Tokyo over the past five years clearly suggests that such dangerous highs in WBGT should be expected for the entire period of the Olympic Games in 2021, with associated increased health risk to both spectators and athletes.

### 4.2. Management of COVID-19 Pandemic

Considering multiple scenarios of COVID-19 [28,29,30], the next summer Olympic and Paralympic Games will be held during the COVID-19 pandemic. While it is not clear whether the number of spectators will be minimized to follow physical distancing rules due to COVID-19, accepting a large number of international travelers from countries experiencing large community transmissions is expected to potentially generate new COVID-19 transmissions due to the increase in social contacts. This ongoing COVID-19 context, including the emergence of novel variants of the virus, clearly suggests that restricting the number of spectators, as argued recently [31], will be indispensable. Although exempting foreign spectators from the 14-day quarantine rule for inbound travelers, and freely allowing their use of public transportation are currently being considered [31], reconsideration of these measures on the latest scientific evidence is required, and should be combined with entry screening. Even for international athletes coming to Tokyo, introducing a contact-tracing app, linking the information with visa and mobility data, and utilizing a large-scale monitoring tool to track self-reported symptoms in real-time [32,33,34] should be considered for promptly detecting potential COVID-19 clusters and facilitating early intervention. While the magnitude of the events is different, several prompt actions in recent operations in other sporting events [35] and their lessons learned should be reflected upon as a reference case.

Additionally, the COVID-19 pandemic has been declared a public health emergency of international concern, and MGEs have great potential to work as an epicenter for infectious disease spread [36]. Therefore, the risk of COVID-19 infection in Japan must be minimized by appropriately combining public health and social measures. This will not only have the benefit of allocating a large number of healthcare resources to border control and the operation of MGEs but for diluting the fear of COVID-19 infection in Japan. Although the ongoing transmission of COVID-19 in Japan has been classified as “clusters of cases” in the World Health Organization (WHO) situation report [37], around 50% of contacts cannot be traced in Tokyo [11], suggesting the presence of some untraced community transmission. Considering the ongoing COVID-19 pandemic and Japan’s overwhelmed public health capacity [1,2,3], securing sufficient capacity for regular COVID-19 screening in multiple venues where the games are scheduled will be a challenge. It must be noted that some Paralympic athletes, who may have underlying conditions, may have higher risk of severe COVID-19, which will fuel a debate on cancellation, re-postponement, or consideration of another location, as discussed in the preparatory phase of the Rio 2016 Olympic and Paralympic Games that were conducted under the threat of Zika virus infection [38,39]. A call for COVID-19 elimination has been emerging in the Japanese context [40], and it is reasonable to raise preparedness for MGEs.

As some participants, including athletes and spectators, will be elderly people and/or people with underlying health conditions, how to use vaccines will be a critical topic. Rapid deployment of vaccines has already started but emerging severe acute respiratory syndrome coronavirus 2 (SARS-CoV-2) variants, their increasing infectiousness and severity, and reduced efficacy of vaccines and vaccine-elicited antibodies for these variants are ongoing scientific challenges that explicitly suggested that vaccines alone will not be able to end the pandemic. Moreover, several challenges in production, affordability, logistics for deployment, and allocation of vaccines should be recognized [41]. Considering the revision of public health recommendations for those vaccinated [42], recommending or mandating vaccination for participants will be considered as one of the contingency plans; however, scenarios should be timely updated in line with latest scientific evidence. Mandatory participation in regular COVID-19 screening testing programs among athletes and spectators will be inevitable. At the same time, however, an open debate about whether prioritizing vaccination for participants of the Olympic and Paralympic Games is truly fair and ethical, in terms of vaccine allocation, is warranted, as even many healthcare workers in low-and middle-income countries are expected to be unable to obtain vaccines with high efficacy in the months and years ahead [43].

Moreover, Japan has lagged in COVID-19 vaccination rates compared to other high-income countries. While vaccination of healthcare providers started in mid-February [44], vaccination for high-risk groups will only begin after April 2021. Relatively low acceptance of the COVID-19 vaccine, multiple issues in health communication during the COVID-19 response, and lessons learned from past vaccine hesitancy in Japan need to be adequately considered [5,21,41,45]. As Japan is expected to return to normality in spring 2022 at the earliest [46], which is after the Olympic and Paralympic Games, building optimistic scenarios depending only on vaccine administration is not a plausible risk management option.

### 4.3. Rethinking Mass Gathering Events from Perspectives in Public Health Systems and Services

Protecting the health of athletes, officials, volunteer staff, and spectators is one of the top priorities when holding MGEs. It must be noted, however, that insufficient risk assessment for health threats for Tokyo 2020 and their impact on public health systems and services was already evident in the preparatory phase, especially when the location of race walks and marathons was suddenly changed from Tokyo to Sapporo, in northern Japan, to protect against heat-related illness [6]. Despite a heat policy presented by the Ministry of Environment [15], most competitions have been scheduled in Tokyo and surrounding prefectures. It is worth noting that the heat index in Tokyo is expected to be much higher than in previous competition venues of the Summer Olympic and Paralympic Games, and the risk of underperformance among potential Olympic athletes who are not acclimatized has been critically argued [23]. Furthermore, many groups with physical disabilities have a higher risk of heat-related illness, but no special health policy has been implemented for Paralympic sports. More rigorous heat regulations and policy are necessary to ensure the health of Paralympic athletes [47]. Even provided that the number of international visitors, which had been assumed to be around 10 million visitors in the pre-COVID-19 era [48], will dramatically decrease, it would be demanding for all visitors to be easily accustomed to the climate in Tokyo, and the number of patients suffering from heat illness in Tokyo will definitely increase. Arranging multilingual health promotion campaigns for preventing heat illness beforehand and organizing health system capacity for their sudden healthcare demands will be vital to mitigate the magnitude of heat illness. Although a coordination meeting for COVID-19 countermeasures at the Olympic and Paralympic Games, which was launched in September 2020, currently argues that COVID-19 is not the sole health threat for the next Olympic and Paralympic Games, there has been little attention to heat policy, which should be recognized as a risk factor for depleting health system capacity in Tokyo and other competition venues. Addressing a shortage of healthcare institutions that can accept foreign patients and provide high quality health services, an overlap with designated medical institutions for COVID-19 [49], and securing enough healthcare workforces as a surge capacity, are essential to ensure sufficient resources for emerging healthcare demands among foreign athletes and tourists, maintain essential health services, and avoid excessive burden on front-line healthcare workers.

The COVID-19 pandemic has affected a wide range of sporting events around the world. Most sporting events have been cancelled or postponed, or held with empty stadiums to ensure the safety of athletes and spectators [50]. Even in 2021, despite rigid quarantine plans and special accommodation plans, many sporting events were still interrupted and/or teams were forced to withdraw; or events themselves were rearranged without fans after detecting positive COVID-19 cases among competitors, as observed at the Australian Open in Australia, the Atlantic Coast Conference (ACC) Tournament in the United States, and the Women’s Rugby World Cup in New Zealand [35,51]. As mass gathering events are associated with the severity of the spread of viral respiratory infectious diseases [52], respecting robust science and evaluating risks will be critical for decision-making, and mitigating these risks by utilizing available resources is important for management [53,54,55]. It should be noted that holding mass gathering sporting events as a pre-planned scenario will potentially bring serious consequences for global sport in the years ahead. If events will be held after the strict health risk assessment, at least revising the scale of spectators to zero or solely allowing residents in Japan with no underlying health conditions to attend the MGEs as spectators under the assumptions that domestic COVID-19 transmission is aggressively suppressed or eliminated, will be reasonable alternatives to a policy of unrestricted access, which risks significant health system pressures from the combined risk of heat illness and COVID-19 outbreaks. To prevent the spread of COVID-19, imposing strict infection control measures for all participants is necessary. Along with this, investing in several devices in competition avenues, such as ventilators, will be required. Although several competitions are scheduled outside Tokyo, managing all events at once will be challenging. Holding some events in COVID-19 eliminated countries or regions separately should also be discussed. Finally, attention to policies on visas, health costs, and international travel consequences of large numbers of visitors being held in COVID-19 treatment facilities after the originally planned expiration of their Olympic travels must be considered, and visitors need to be adequately informed in their country of origin of the potential financial and personal risks associated with prolonged treatment for heat-related illness and/or COVID-19.

In summary, our report suggests that the period of the next Olympic and Paralympic Games in Tokyo in 2021 will potentially coincide with the peak of emergency transportations due to heat illnesses, and the number of those will increase in line with possible inbound visitors and long-term trends of heat risk in Tokyo. This would be a major blow to maintaining a responsive health system, especially emergency medical services, which have already been overwhelmed by the COVID-19 pandemic. Without implementing countermeasures for all health threats based on the latest evidence, the health system capacity in Tokyo will be overwhelmed by excessive burden, and maintenance of essential health services will be again interrupted during the Olympic and Paralympic Games in July–September 2021. To protect the health of those involved in MGEs, maintain essential health services, and ensure access to healthcare of citizens, further urgent consideration of the timeframe [56] and competition venues needs to be given based on the robust science. As the COVID-19 pandemic will not end until it is over everywhere, global solidarity for containing the virus based on excellent science and innovative investment will be critical. Reforming Japan’s overwhelmed public health interventions, such as a continuum of testing, tracing, and isolation, will be indispensable. Moreover, imposing quarantine for returning athletes in their home countries depending on their vaccination history could help minimize the risk of re-sparking local outbreaks, thus contributing to destigmatizing Tokyo 2020 as a COVID-19 exportation hub. Simultaneously, however, there are still many uncertainties in the transmission dynamics of COVID-19 at a global scale; therefore, sparing flexible rooms for modifications, restriction, re-postponement, and cancellation of the events need to be secured. Ensuring openness and transparency for health risk assessment will be vindicated by preventing the Tokyo Olympic and Paralympic Games from becoming a global super-spreader event.

### 4.4. Limitations

Several limitations should be noted. First, this report relied upon descriptive statistics and mainly discussed the epidemiology of heat illness and COVID-19, and health system capacity in terms of emergency medical services only in Tokyo; however, as mentioned above, there are some competition venues outside Tokyo [6]. While these may mitigate the overall impact of potential health threats in Tokyo, it will inevitably mean that raising preparedness for additional healthcare demands brought by athletes, officials, volunteer staff, and spectators must be considered in local regions. A more detailed scenario regarding the number of incoming visitors and spectators for each event will be necessary to estimate the potential healthcare demands during the next summer Olympic and Paralympic Games. As many of these areas have a large proportion of elderly local residents, extra protection against outbreaks of COVID-19 will be necessary. Second, our data regarding heat illness only included the transferred cases by ambulance and did not include walk-in patients. As some walk-in–heat illness patients require admission, the impact on hospital capacity might have been marginally undervalued. Third, careful interpretation is needed for Tokyo rule, as the increase of nosocomial infections of COVID-19 made more healthcare workers self-quarantine, thus contributing to decreasing the effective hospital capacity and increasing rejections of ambulances. Fourth, we could not calculate the proportion of the Tokyo rule transportations among all emergency transportations in 2020, though they rose from 0.98% in 2018 to 1.27% in 2019 [57], as the number of all emergency transportations per day and week is not available to the public, and the yearly data have not been published yet. While we assume that the proportion has increased in 2020, as overall dispatches of ambulances decreased by more than 100,000 [58], more detailed data are warranted to support our premise.

## 5. Conclusions: Implications for the Summer Olympic and Paralympic Games in 2021

The COVID-19 pandemic posed a host of issues in maintaining essential health services, including emergency medical services. Although COVID-19 has emerged as a monumental health threat for holding mass gathering events, it should not be conceptualized as a sole threat. Heat illness along with increasing heat stress index is definitely one of the large health threats for MGEs in Tokyo, especially among the elderly, and an overstretched health system capacity need to be comprehensively discussed through an all-hazards approach. Without this, healthcare capacity will be overwhelmed by excessive healthcare demand, thus challenging maintenance of essential healthcare services and putting additional burden on front-line health-care workers. Our results can be utilized as underlying information for openly conducting a health risk assessment, developing multiple scenarios regarding the number of spectators for MGEs and COVID-19 situations, and leveraging them with the health system capacity in the preparatory phase of the next summer Olympic and Paralympic Games in Tokyo in 2021. Ensuring openness and transparency for health risk assessment will be critical to prevent irreparable impact on the trajectory of COVID-19 and global sports during this global public health emergency.

## Figures and Tables

**Table 1:** Number of emergency transportations due to heat illness in Tokyo in weeks 29–36 in Tokyo in 2016–2020, by age and severity.

		Year				
		2016	2017	2018	2019	2020
**Age** **(years)**	0–17	10.3% (185)	9.9% (182)	9.7% (605)	7.3% (369)	5.4% (264)
	18–64	38.7% (692)	42.5% (778)	43.6% (2736)	38.8% (1959)	36.8% (1787)
	65-	51.0% (912)	47.6% (871)	46.7% (2928)	53.9% (2720)	57.8% (2811)
**Severity**	mild	61.9% (1107)	61.4% (1125)	61.7% (3865)	58.0% (2928)	57.4% (2789)
	moderate	36.5% (653)	36.4% (666)	35.0% (2193)	36.9% (1864)	37.9% (1842)
	severe/fatal	1.6% (29)	2.2% (40)	3.3% (209)	5.1% (255)	4.8% (231)
**Total**		1789	1831	6269 *	5048 *	4862

* As the severity of 2 heat illness transportations in 2018 and 1 in 2019 was classified as “others” whose severity or diagnosis remained unknown or who were transferred to other places, the total number did not correspond to the sum of the three categories.

## Data Availability

The data are publicly available at websites cited on references [11,12,17].
